# Artificial intelligence in Chinese healthcare: a review of applications and future prospects

**DOI:** 10.1007/s13534-025-00515-2

**Published:** 2025-10-23

**Authors:** Zihuan Wang

**Affiliations:** 1 National Engineering Research Center of Ophthalmology and Optometry, Eye Hospital, Wenzhou Medical University, Wenzhou, China; 2 Center of Optometry International Innovation of Wenzhou, China Eye Valley, Wenzhou, China; 3 Institute of Engineering Research, Seoul National University, Seoul, South Korea

**Keywords:** Artificial intelligence, Healthcare, China, Medical devices, Regulation, Telemedicine

## Abstract

China’s healthcare infrastructure faces growing population pressure and resource gaps. This review explores how AI applications, regulatory frameworks, and commercialization pathways are reshaping China’s healthcare delivery system and global innovation standards. China’s AI healthcare market is expected to grow from $900 million in 2020 to $1.59 billion in 2023, and is expected to reach $18.88 billion by 2030, at a Compound Annual Growth Rate (CAGR) of 42.5%. The National Medical Products Administration (NMPA) expects to approve 59 Class III AI devices by 2023, compared with just nine in 2020. Key applications include the widespread use of AI technology in lesion identification; a telemedicine platform serving 13 million users; and AI drug development that shortens the development cycle from 4 to 18 months. Regulatory pillars include the Personal Information Protection Law, which requires explicit consent before processing health data, and NMPA guidelines, which require all AI medical software to undergo three types of review. China’s unique combination of centralized health data, policy incentives, and rapid commercialization has created a globally competitive AI medical ecosystem. Continued development requires addressing issues such as algorithm transparency, cross-border data governance, and international regulatory coordination.

## Introduction

China’s healthcare system serves a population of 1.4 billion and faces the pressure of a demographic transition, with an estimated 400 million elderly people by 2035 [1]. The traditional hospital-centric model faces a shortage of doctors, with misdiagnosis rates as high as 30–40% at primary care facilities [1]. The Chinese government’s “Healthy China 2030” plan sees AI as a transformative technology that aims to improve diagnostic accuracy, optimize resource allocation, and expand access to healthcare [2].

The trajectory of China’s AI healthcare market reflects both policy ambition and practical need. Market valuations have soared from $900 million in 2020 to $1.59 billion in 2023, and are expected to reach $18.88 billion by 2030, a compound annual growth rate of 42.5% [3, 4]. This expansion is occurring within a supportive ecosystem that includes centralized electronic health records, regulatory innovation pilots, and significant government investment in AI development, totalling $1.4 trillion by 2030 [3–6].

The regulatory landscape strikes a balance between encouraging innovation (through fast-track and sandbox pilots) and ensuring patient safety (through strict Class III medical device requirements and PIPL data protection standards [7, 8]). This approach provides a model for other healthcare systems to adopt AI while maintaining clinical oversight and ethical compliance.

We conducted a comprehensive literature search in PubMed, Web of Science, China National Knowledge Infrastructure (CNKI), and regulatory portals including the National Medical Products Administration and Cyberspace Administration databases. The search terms combined English keywords (“China AI healthcare”, “NMPA medical device”, “telemedicine China”) and Chinese keywords ("人工智能医疗," "药监局," "远程医疗"). The search spanned January 2018 to June 2025 to capture the latest policy developments and market evolution.

## Market landscape and growth dynamics

Driven by strategic policy initiatives, an aging population, and rapid technological advancements, the valuation of China’s AI healthcare industry is expected to expand from $900 million in 2020 to $1.59 billion in 2023, with a three-year compound annual growth rate of approximately 22% [3]. Forecasts show that the market will continue to grow exponentially, reaching US$7.33 billion in 2028 and US$18.88 billion in 2030, respectively, with a compound annual growth rate of 42.5% [4]. This expansion has been supported by heavy government investment through the National AI Strategy, which aims to achieve a core AI industry value of US$68.97 billion, with healthcare listed as one of the top five priority application areas [2]. Regional innovation hubs such as Beijing (market share of 19.3%), Shanghai (market share of 14.6%), and Shenzhen offer regulatory sandboxes, tax incentives, and shared research infrastructure [2]. Venture capital has flowed to these cities, and more than 150 AI healthcare companies are competing in the imaging, drug development, and telemedicine verticals.

The market structure reflects China’s healthcare focus, with medical imaging accounting for the largest share, with a cancer detection market size of Chinese Yuan (CNY) 300 million and a lung nodule detection market size of CNY 250 million [2]. AI drug discovery is the fastest growing area, with more than 100 pharmaceutical companies founded since 2018 focusing on AI-driven compound identification and optimization [9].

## Core AI applications in Chinese healthcare

Several studies have shown that China’s CT and MRI inventory, measured per capita, has long been below the OECD median, with a pronounced “high in the east, low in the middle, and even lower in the west” pattern. Equipment allocation is positively correlated with GDP, the number of hospitals, beds, and the number of health workers, suggesting that economic and supply factors are driving the concentration of high-end imaging resources in developed regions. Although the Gini coefficient for CT and MRI declined overall and distribution improved between 2005 and 2013, the concentration index remained positive (≤ 0.30), indicating that relative disparities have persisted. More recent data show that the number of imaging devices per million people has increased rapidly over the past two decades, but regional disparities persist [10–12].

Insufficient primary care diagnostic and continuity of care capacity is confirmed by multiple sources of evidence. Based on nationally representative tracking data (2011–2018), the probability of visiting village clinics decreased by 44%, with patients shifting to county-level hospital outpatient clinics (+ 56%) and self-medication (+ 20%), reflecting the dual constraints of primary care accessibility and service capacity/trust. Comprehensive reviews also point to structural issues within the primary care system, such as uneven infrastructure development, declining medical insurance funding, and insufficient incentives. Multi-center measurements in 2025 show that the quality of primary care (PCAT) at primary care facilities remains low, particularly in dimensions such as “community orientation.” These evidences collectively point to rising demand for imaging and chronic disease management at the grassroots level, but the supply (including personnel, equipment, and processes) has not kept pace [13–15].

Currently, China has entered a period of rapid aging: in 2019, the population aged 60 and above was approximately 254 million; by 2040, it is expected to be approximately 402 million, accounting for approximately 28% of the total population; in 2021, the proportion of people aged 65 and above reached 14.2% and continues to rise. Aging will significantly increase the demand for diagnosis and follow-up related to chronic diseases, rehabilitation, long-term care, and cognitive impairment, placing continuous pressure on the entire medical system [16–18]. Relevant literature shows that the application of AI in China's medical field can make up for resource imbalances, improve efficiency and affordability; regulatory authorities have also approved a few AI medical devices, providing a path for clinical implementation [1, 19].

In summary, it is understandable why China needs to increase its investment in AI in the medical and healthcare fields. Against the backdrop of uneven spatial distribution of high-end imaging resources, insufficient grassroots service capabilities, and a surge in demand driven by aging, AI can serve as a “supply-side enhancer”: first, imaging AI assists in triage and quality control, alleviating bottlenecks in film reading, shortening waiting times, and reducing misdiagnoses; second, through AI-enabled remote consultations, follow-up, and screening in grassroots scenarios, accessibility and consistency can be improved; third, process and resource optimization (such as intelligent scheduling and predictive capacity management) can improve system efficiency.

### Medical imaging and diagnostics

Medical imaging dominates AI healthcare deployment in China, accounting for 67% of the 59 Class III medical devices approved by the National Medical Products Administration by 2023 [7]. Deep learning algorithms can automatically detect pulmonary nodules, intracranial hemorrhage, and coronary artery stenosis, and clinical trials have shown that the interpretation time of chest CT scans has been reduced from 10 min to 30 s, while improving sensitivity by 9 percentage points [20].

Clinical validation studies have shown that the system has strong performance in multiple imaging modes. A multicentre trial of the Shukun CT post-processing system achieved an AUC of 0.848, 82.9% sensitivity, and 88.5% specificity in 88 patients [20]. The thyroid ultrasound AI system developed by Zhejiang University and deployed in multiple hospitals in China has improved the accuracy of radiologists by 10% in the process of processing more than 5,000 patients [20].

Tencent’s AI medical imaging platform “Tencent Miying” has achieved an accuracy of 90% in early esophageal cancer screening in clinical validation in more than 100 large hospitals [21]. The system has demonstrated the ability to detect diabetic retinopathy (with 97% accuracy) and colorectal adenomas, with endoscopic screening completed in 4 s [22, 23].

Leading domestic companies such as Shukun Technology (with 9 NMPA approvals) and Shenzhen Zhihui Medical (with 6 approvals) have achieved technical parity with international competitors [7]. Cancer detection applications have huge market value, with AI-assisted tumor imaging valued at CNY 300 million and lung nodule detection valued at CNY 250 million [2].

### Telemedicine and virtual care

China’s telemedicine industry is incorporating AI into patient triage, consultation, and post-consultation monitoring. Company “Ping An Health Insurance” operates a leading platform in China with 373 million registered users, powered by AI avatars that mimic the voice and clinical reasoning of specialist doctors [24]. The system interprets lab results with 98% accuracy and provides 24/7 consultation services through text, voice, and video interfaces [24]. Ping An’s “Xin Yi” service uses digital twins of real doctors and is built on three layers of training: (1) basic medical knowledge from five databases covering 37,000 diseases and 420,000 terms; (2) personal doctor knowledge including publications and social media content; and (3) personalized fine-tuning through direct training of doctors [25]. The platform has a triage accuracy of over 99% and an auxiliary diagnosis accuracy of over 95% [26]. Ping An’s “One Minute Clinic”—a 3-square-meter automated medical self-service terminal that can perform AI-powered diagnosis of more than 100 common diseases—is planned to be rolled out in transportation hubs and townships across the country [27]. This service model demonstrates how AI technology can narrow the gap in medical resources between cities and towns by providing standardized and convenient basic medical services.

Tencent’s WeChat Health Mini Program serves more than 80 million users, providing services such as appointment booking, health check-ups, vaccinations, and online pharmacy inquiries [28]. Based on user characteristics, the platform combines customized health education services with personalized content push services based on user preferences and medical history.

### Smart hospitals and AI agents

Tsinghua University’s AI-powered Agent Hospital exemplifies the cutting edge of AI in medicine. Housing 42 autonomous “AI doctors” across 21 specialties, it recreates every step of patient care—from initial triage and diagnosis through treatment planning and follow-up—in a fully virtual environment. In validation studies, these AI clinicians achieved a remarkable 93.06% diagnostic accuracy on China’s medical licensing examination, on par with practicing physicians. Even more striking, in simulation trials a single AI doctor processed 10,000 virtual cases in just days—equivalent to two years of patient encounters for a human practitioner.

Underlying this capability is the MedAgent-Zero framework, which forgoes traditional, manually labeled datasets in favor of self-supervised learning through patient-agent interactions. This design accelerates skill acquisition and model refinement, enabling the AI to adapt rapidly to new clinical scenarios [29].

While the diagnostic performance and scalability of Agent Hospital are impressive, its ultimate value will depend on seamless collaboration with human clinicians, robust safety monitoring, and clear regulatory pathways. If successfully deployed, this model could dramatically expand access to specialized expertise, particularly in under-resourced regions—and set new benchmarks for how AI augments rather than replaces human judgment in healthcare (Tables 1, 2 and 3).

**Table 1:** AI medical market size in 2020–2030

Year	Market value (USD billion)	NMPA AI device Approvals	Key policy milestones	References
2020	0.90	9	First AI device license	[3]
2023	1.59	59	PIPL enforcement begins	[7]
2028	7.33	–	CMDE No. 38 guidance implementation	[31]
2030	18.88	–	High-end Device Measures	[4]

**Table 2:** AI application scenarios

Application	Performance metrics	Sample size/deployment	References
CT lung nodule detection	0.848 AUC, 82.9% sensitivity	88 patients, multicenter	[20]
Thyroid ultrasound AI	10% improvement over radiologists	5,000 + patients	[20]
Esophageal cancer screening	90% accuracy rate	> 100 hospitals validation	[21]
IPF drug discovery	+ 98.4 mL FVC vs -20.3 mL placebo	71 patients, 21 sites	[49]
Telemedicine consultations	98% lab interpretation accuracy	13 million subscribers	[24]

**Table 3:** AI compliance policy summary

Regulation	Scope	Key healthcare provisions	Year	References
PIPL	Personal data protection	Explicit consent, cross-border restrictions	2021	[8]
Cybersecurity Law	Network operations	Data localization for critical infrastructure	2017	[48]
Data Security Law	Important data classification	Risk-based export controls	2021	[48]
CMDE No. 38	AI diagnostic software	Clinical trial metrics, transparency	2023	[31]
High-End Device Measures	Rapid Review	Conditional approvals	2025	[32]

Operational AI optimizes hospital workflows through predictive analytics for operating room scheduling, equipment maintenance, and patient flow management. Early pilot studies have shown that average length of stay can be reduced by 8% through AI-optimized resource allocation [30].

## Regulatory framework and policy environment

### NMPA device classification and approval

China’s National Medical Products Administration classifies all AI medical software as Class III medical devices, which are subject to the most stringent regulatory review process. This is in stark contrast to the US Food and Drug Administration (FDA). Currently, 96% of AI devices approved by the FDA are classified as Class II, reflecting China’s cautious attitude towards algorithm-driven healthcare applications at this stage [7]. China’s 2023 Medical Device Design and Design Guidelines No. 38 standardize clinical evaluation requirements and specifies specific performance metrics (AUC, F1 score) and sample size calculations for AI diagnostic software [31].

NMPA approvals increased from 9 devices (2020) to 59 devices (2023), with radiology applications comprising 67% of approvals[7]. Seven devices hold dual FDA-NMPA clearance, though U.S. approval typically precedes Chinese approval by 8–14 months due to different regulatory pathways[7]. Change registration procedures were simplified in 2023 for AI devices where core algorithms remain unchanged, facilitating post-market iteration[32].

### Data privacy protection

PIPL, which came into effect in November 2021, marks the establishment of a comprehensive data privacy framework in China [8]. PIPL requires explicit consent for the processing of health data and strengthens the protection of “sensitive personal information”, including medical records, genetic data, and biometric information [8, 33]. If data needs to be transferred across borders, it is required to undergo a security assessment or a standard contract filing by the Cyberspace Administration of China, which to some extent imposes compliance constraints on multinational medical AI companies.

The latest regulatory policy update in 2024 has relaxed the transfer threshold and increased the limit on the number of non-sensitive personal information that triggers a security assessment from 100,000 to 1 million [8]. However, any sensitive health data transfer requires a separate assessment, thereby strictly regulating medical AI data flows [34]. A compliance audit of 30 provincial health code applications showed that the average compliance rate for PIPL was 59.9%, with only one application obtaining separate consent for sensitive data processing [34]. This shows that despite regulatory requirements; the implementation of the policy still faces significant challenges.

### Innovation incentives and rapid review

China has implemented targeted policies to accelerate the commercialization of AI medical technology while ensuring safety standards. The 2025 Measures for the Administration of High-end Medical Devices establishes a conditional approval pathway for AI innovation projects pioneered in China, allowing them to enter the market and requiring ongoing post-market surveillance [32]. The free trade zones in Shanghai, Beijing, and Hainan offer regulatory sandboxes to promote international cooperation and technology transfer pilots [35]. The Shanghai Zhangjiang AI Island provides rental subsidies and shared GMP laboratories for startups that obtain clinical trial approval from the National Medical Products Administration (NMPA) [35, 36]. The 66,000 square meter facility houses not only global companies such as IBM, Microsoft, and Alibaba, but also domestic AI companies [35, 37]. These innovation zones have accelerated industry-university-research collaboration and facilitated universities to license AI technologies to commercial partners for clinical applications.

### Technology transfer and industrialization

Chinese AI healthcare companies increasingly adopt “AI-as-a-Service” revenue models, transitioning from one-time software licenses to subscription-based offerings averaging USD 30,000 annually per tertiary hospital installation [27]. This shift enables continuous algorithm improvement through real-world data collection while providing predictable revenue streams for developers.

Academic translation mechanisms flourish through dedicated innovation parks and university-industry partnerships. Tsinghua’s AIR laboratory licenses its MedAgent-Zero technology to commercial spin-offs for hospital deployment, bundling cloud computing infrastructure with AI model updates [29]. Similar partnerships between Beijing, Shanghai, and Shenzhen universities channel research breakthroughs into clinical applications through structured commercialization pathways.

International collaboration proceeds despite cross-border data constraints, with Chinese companies establishing overseas research centers and joint ventures. XtalPi maintains laboratories in Boston and partnerships with MIT and Singapore’s A*STAR, enabling access to global talent and regulatory expertise while complying with Chinese data localization requirements [38].

Federated learning frameworks offer solutions to data sharing constraints while enabling multi-institutional model training. The Contribution-Aware Federated Learning (CAFL) framework deployed by Yidu Cloud Technology serves 8 medical institutions in China, performing contribution evaluations 2.84 times faster than existing approaches while improving model accuracy by 2.62% [39]. Federated learning market projections indicate growth from USD 2.7 million (2024) to USD 5.9 million (2030) in China [40].

### International collaboration and global impact

China engages actively in global AI healthcare governance through WHO-ITU “AI for Health” initiatives, contributing radiology datasets for international algorithm benchmarking [41, 42]. The Global Initiative on AI for Health, launched jointly by WHO, ITU, and WIPO in 2023, includes Chinese participation in establishing standardized assessment frameworks for AI-based health interventions [41, 43].

Chinese telemedicine technologies expand internationally through Belt and Road health accords, with platforms like Ping an Health exporting AI-enabled consultation systems to partner nations [27]. These deployments enhance China’s soft power influence while providing revenue diversification for domestic companies.

Regulatory harmonization efforts face challenges due to divergent classification approaches between Chinese (Class III), U.S. (Class II), and European (CE marking) systems [7]. However, seven AI devices currently hold dual approvals, indicating potential pathways for international regulatory alignment [7]. The WHO’s ethics and governance guidance for AI in health, developed with Chinese input, provides frameworks for responsible AI deployment globally [41].

## Current challenges and limitations

### Algorithm transparency and explainability

Proprietary AI algorithms pose fundamental challenges to clinical adoption and regulatory compliance. Healthcare providers express concerns about “black box” decision-making systems that lack interpretable outputs, hindering physician trust and patient acceptance [7]. The 2023 CMDE guidelines now request explainability annexes for AI device submissions, though implementation standards remain unclear [31].

Medical liability frameworks struggle to address AI-assisted decision-making, particularly when algorithm recommendations conflict with physician judgment. Legal scholarship advocates for patient-centric information disclosure standards and restructured liability rules accommodating human-AI collaboration in clinical settings [44].

### Data fragmentation and interoperability

Despite national electronic health record initiatives, data silos persist across China’s 34 provincial healthcare systems, limiting AI model portability and training data quality [45]. Inconsistent data standards and varying hospital information systems create technical barriers to large-scale AI deployment. PIPL requirements for explicit consent and purpose limitation further complicate multi-institutional data sharing essential for robust AI training datasets [34]. Federated learning approaches offer potential solutions but lack standardized implementation frameworks [44]. Only 5.2% of 612 federated learning studies achieved real-world deployment, indicating substantial technical and regulatory barriers [44].

Training data concentrations in urban tertiary hospitals risk algorithm bias against rural and minority populations [46]. Medical AI systems optimized for well-resourced hospital settings may underperform in primary care environments serving China’s 500 million rural residents [1]. Generative AI applications face additional ethical challenges regarding information accuracy, patient privacy, and clinical decision-making autonomy [47]. Healthcare institutions require comprehensive ethical frameworks addressing AI bias mitigation, transparency requirements, and human oversight protocols [47].

Fundamental differences between Chinese Class III designation and international Class II/CE marking approaches create substantial barriers for multinational AI healthcare companies [7]. Dual approval processes require separate clinical trials, regulatory submissions, and quality management systems, increasing development costs and market entry timelines. Data localization requirements under PIPL and Data Security Law constrain international collaboration in AI model development, particularly for companies requiring global datasets for algorithm training and validation [8, 48]. Regulatory uncertainty regarding cross-border AI model deployment impedes multinational healthcare technology investments.

## Discussion and conclusion

China’s AI healthcare ecosystem exemplifies how coordinated policy support, centralized data resources, and market incentives can synergistically accelerate innovation adoption. The confluence of the Personal Information Protection Law (PIPL), the Data Security Law, and fast-track regulatory sandboxes has underpinned a 42.5% market CAGR and rapid growth in Class III AI device approvals—from 9 in 2020 to 59 in 2023—while unified electronic health records and RMB 1.4 trillion in government investment have fueled the market’s expansion from USD 1.59 billion to a projected USD 18.88 billion by 2030. These systemic advantages have enabled large-scale deployments across medical imaging, drug discovery, telemedicine platforms, and AI-powered smart hospitals, demonstrating China’s capacity to translate policy into clinical impact.

Achieving sustainable, equitable AI-driven healthcare at scale requires addressing several interrelated challenges. First, as AI systems assume more advanced diagnostic and treatment roles, algorithm explainability must be strengthened through transparent model reporting, post-market performance monitoring, and integration of explainable AI techniques—such as saliency mapping and counterfactual analysis—to maintain clinician trust and patient safety. Second, healthcare equity demands expanding training datasets beyond urban tertiary centers to include rural, minority, and community-level patient cohorts, ensuring algorithms perform robustly across diverse demographic groups and reducing the risk of algorithmic bias. Third, data privacy and sovereignty under PIPL necessitate federated-learning and other privacy-preserving frameworks that allow multi-institutional model training without centralizing sensitive health data, preserving security while enabling collaborative innovation. Fourth, international regulatory harmonization—through active participation in WHO-ITU AI4 Health governance initiatives and bilateral research consortia—can align performance standards, streamline dual approvals, and foster global diffusion of best practices.

Looking ahead, prospective clinical outcome studies will be crucial to quantify AI’s real-world impact on key metrics such as mortality reduction, hospital readmission rates, patient satisfaction, and cost-effectiveness. Rigorous health economics analyses and multicenter randomized trials will provide the evidence base needed for adaptive regulatory policies and reimbursement frameworks, guiding iterative algorithm refinement and informing transferable best practices for global healthcare systems. Furthermore, continued investment in clinician AI-literacy programs and interdisciplinary collaboration will be essential to integrate AI as an augmentative tool—rather than a replacement—thereby enhancing clinical decision-making and delivering value across the entire care continuum.
